# Changes in characteristics of veterans using the VHA health care system between 1996 and 1999

**DOI:** 10.1186/1478-4505-3-5

**Published:** 2005-04-18

**Authors:** Chuan-Fen Liu, Matthew L Maciejewski, Anne EB Sales

**Affiliations:** 1 Department of Veterans Affairs, Puget Sound Health Care System, Health Services Research and Development, USA; 2 University of Washington, School of Public Health, USA

## Abstract

**Background:**

The Department of Veterans Affairs' Veterans Health Administration (VHA) provides a health care safety net to veterans. This study examined changes in characteristics of veterans using the VHA health care system between 1996 and 1999 when VHA implemented major organizational changes to improve access of ambulatory care and to provide care to more veterans.

**Methods:**

The study used two cross-sectional samples of the Medical Expenditures Panel Survey (MEPS), a national representative survey, in 1996 and 1999. The 1996 MEPS survey included 1,944 veterans and the 1999 MEPS survey included 1,974 veterans. There were 534 veterans and 740 veterans who used VHA services in 1996 and 1999, respectively.

**Results:**

The proportion of veterans using the VHA system increased from 12.4% in 1996 to 14.6% in 1999. In both years, veterans were more likely to use VHA care if they were older, male, less educated, uninsured, unemployed, and in fair or poor health status. Only two variables, marital status and income, were different between the two years. Married veterans were more likely to use VHA care in 1999, but not in 1996. Veterans with higher incomes had greater odds of using VHA care in 1996, but there was no significant association between income and VHA use in 1999.

**Conclusion:**

Characteristics of VHA users did not fundamentally change despite the reorganization of VHA health care delivery system and changes in eligibility and enrollment policy. The VHA system maintains its safety net mission while attracting more veterans.

## Background

Veterans have access to a health care system unavailable to most Americans – the Veterans Health Administration (VHA), part of the Department of Veterans Affairs. This large, integrated health care system provided care to about 4.5 million veterans in 2002. In 2001, the total veteran population was estimated at 25.3 million, accounting for about 10% of the U.S. total population [1].

The VHA has implemented significant organizational changes in recent years as mandated by the Veterans Eligibility Reform Act of 1996 [2]. The reorganization of the VHA aimed to shift the focus of care from inpatient to outpatient settings, to improve access to ambulatory care, and to provide care to more veterans. Prior to 1996, only inpatient care was mandated and outpatient care could not be provided unless there was an antecedent inpatient admission for the same problem. Legislative changes were necessary to allow VHA practitioners to provide more ambulatory care when appropriate and to free up inpatient resources that could be used to care for more veterans in outpatient settings [3].

While the VHA was shifting from inpatient to outpatient care, the VHA system was also reorganized from four large regional units into 22 Veterans Integrated Service Networks (VISNs), which became the primary budgetary and organizational unit for the health care facilities in each network. As of 2003, there are 21 VISNs. In addition, VHA was allowed by Congress to establish Community-Based Outpatient Clinics (CBOCs), linked to existing local medical centers, to improve access for veterans in suburban and rural areas [4,5]. The number of CBOCs increased from 10 in 1996 to 228 in 1999 [5], and to over 800 in 2003 [6]. VHA has transformed from a confederation of individual medical centers and clinics focused primarily on inpatient care to a fully integrated health care system that promotes primary and ambulatory care [7]. Over the 1996–2000 period, the annual number of ambulatory care visits has increased by 35%, the number of acute care hospital beds has dropped by 35%, inpatient admissions have dropped by 36%, and total bed days of care have been reduced by 68% [2].

Organizational reforms were accompanied by VHA eligibility and enrollment changes. The VHA system's historic mission has primarily been to serve as a safety net for veterans with service-related disability or low income [2]. In 1999, the VHA system was opened to all veterans based on a new enrollment system based on the Veterans Eligibility Reform Act of 1996. In the current eligibility system, vulnerable veterans who have been eligible to receive free care continue to receive free care. For many of these veterans, the VHA continues to represent an important safety net because many of them are indigent and/or vulnerable. A new group of veterans have been receiving VHA care for the first time in large numbers, including veterans without service connected disability, veterans whose level of service connected disability does not entitle them to compensation and their income is above the VHA means test threshold. These (Priority Group 7) veterans are required to make co-payments for certain types of VHA care.

On October 1, 1998, the VHA began an enrollment program that registered veterans using the health care system and assigned them a primary care provider. The enrollment program and other changes were implemented to support two goals established in the 1998–2003 Strategic Plan for the Department of Veterans Affairs: 1) to increase the number of users of the VHA health care system by 20% by 2003, and 2) to increase the percent of the operating budget obtained from revenues sources that are not appropriated by Congress, to 10% of the total [8]. VHA facilities were encouraged to generate additional revenues from sources other than Congressional appropriations and took two approaches to obtaining non-appropriated revenues. First, VHA facilities have improved billing to private insurers for veterans with private insurance while using VHA care [9]. The second approach was to attract more insured veterans. Non-elderly veterans with insurance are likely to be different from the historic population of veteran users of VHA care, because many are likely to be healthy enough to be employed and insured. In addition to generating non-appropriated revenues, increased enrollment of insured non-elderly veterans may change the overall risk profile of VHA users. Elderly veterans with insurance are almost all enrolled in Medicare, and may or may not be healthier than existing users of VHA care. The VHA has had to balance the conflicting challenges of continuing its health care safety net mission and, at the same time, attracting new users with insurance who can generate non-appropriated revenues.

Prior to these eligibility, enrollment and organizational changes, veterans seeking care at the VHA were known to be sicker than the general population [10,11]. The policies and organizational changes may attract a broader group of veterans entering the VHA system. The VHA has been a safety net for many indigent veterans and could be a new source of care for the 90% of veterans who do not seek VHA care in a given year [10,12]. In prior studies, researchers have examined VHA user or enrollee characteristics from administrative databases or the Survey of VA Enrollees [12,13]. However, there is to date no research assessing changes in veteran characteristics between veterans who use VHA services (VHA users) and those who do not use VHA services (VHA nonusers) during the period 1996–1999, with its major policy and organizational changes in the VHA.

In this study, we assess whether there were changes in demographic characteristics, health insurance coverage, and health status among veterans who used the VHA system between 1996 and 1999 when VHA underwent major organizational changes in health care delivery using the Medical Expenditure Panel Survey (MEPS). We hypothesize that these policy changes, especially the change in the enrollment policy, would attract more insured and healthier veterans to the VHA system. MEPS data, comprised of a nationally representative sample of the civilian, non-institutionalized U.S. population data, provides a unique opportunity to examine the characteristics of VHA users and VHA nonusers among veterans.

## Methods

This analysis compared the characteristics of veterans who used VHA care and those who did not use VHA care using two cross-sectional samples in MEPS in 1996 and 1999.

### Data Sources

The data sources were the 1996 and 1999 Household Component (HC) of the MEPS, which is co-sponsored by the Agency for Healthcare Research and Quality and the National Center for Health Statistics. We used the 1996 and 1999 Full Year Consolidated Data Files from the MEPS HC survey, which contained questions on demographic characteristics, health conditions, health status, having usual source of care, health insurance coverage, income, and employment status. The files also captured information about use and expenditures of medical services for office and hospital-based care, home health care, dental services, vision aids, and prescribed medicines. The two files covered the calendar years 1996 and 1999, respectively. For a detailed description of the MEPS sampling and survey methods, see Cohen [14].

### Study Sample

Survey respondents were identified as veterans by answering affirmatively to the following question: "Did you ever serve in the Armed Forces?" The MEPS surveys included 1,944 veterans in 1996 and 1,974 veterans in 1999. The number of veterans represented 9.2% and 8.5% of the MEPS sample for 1996 and 1999, consisting of 26.6 and 25.7 million veterans in 1996 and 1999, respectively. These estimates are similar to the Census estimates, which were 26.5 million in 1996 and 26.1 million in 1999 [15,16].

### Variable Definitions

#### VHA User and VHA Nonuser

VHA users were defined as veterans who used VHA care in 1996 and 1999, respectively. We used MEPS expenditure data to identify VHA users, based on non-zero medical expenditures paid by VHA facilities in 1996 or 1999. VHA nonusers were defined as veterans who did not have any medical expenditure paid by VHA. The definition of VHA users we used in this analysis is similar to the one used by VHA administratively to allocate resources among VHA facilities. There were 534 and 740 VHA users among veterans in the 1996 and 1999 MEPS surveys, respectively.

#### Health Insurance Status

Veterans were defined as entirely insured if they had either public or private insurance coverage that included both hospital and physician care throughout the entire year for 1996 and 1999, respectively. Veterans were defined as partially uninsured if they had insurance coverage for some segment of 1996 or 1999, or entirely uninsured in a year. Uninsured veterans were split about half partially uninsured and half entirely uninsured. We combined partially uninsured and entirely uninsured veterans into the uninsured group because two groups of veterans had similar characteristics.

#### Health Status

Two measures, perceived physical health status and mental health status, were used. Both health status measures were rated as excellent, very good, good, fair, or poor.

#### Employment Status

Veterans were categorized into three employment status groups – unemployed, retired, and employed. Veterans were defined as unemployed if they were unemployed throughout one entire year; retired veterans were those who did not work in a year due to retirement; and employed veterans were defined as those who worked at least for a part of a year.

#### Usual Source of Care

Veterans were defined as having a usual source of care if they answered yes on the question "Did you have a usual source of care provider?"

### Population Weights

Additional adjustment to the MEPS population weights from the full year sample are needed to accurately estimate VHA users and VHA nonusers, because the MEPS weights do not take into account the status of VHA user versus nonuser. MEPS would allow us to generate accurate estimates for the national population of veterans for December 31, 1996 and December 31, 1999, but not for VHA users and VHA nonusers. Therefore, we applied three additional adjustments to the MEPS population weights. First, we adjusted the proportions of VHA users and nonusers in 1996 and 1999, respectively. The MEPS overestimated the numbers of VHA users and underestimated the number of VHA nonusers. Based on MEPS, there were 7.2 million VHA users in 1996 and 9.8 million in 1999, while there were 2.9 million users in 1996 and 3.4 million in 1999 based on VHA workload data [18]. Second, we adjusted the age distribution of veterans based on the Census data, because the MEPS over-weighted veterans under 55 years old and under-weighted those 55 or older. Third, we adjusted the age distribution of VHA users based on the age distribution of VHA users, because the MEPS under weighted VHA users under 55 years old, and over weighted VHA users 55 or older.

### Statistical Analysis

To examine the differences in socio-demographic characteristics, health status, and having usual source of care between VHA users and non-users in 1996 and 1999, as well as among VHA users between 1996 and 1999, we used bivariate analyses that were conducted using Pearson Chi-Square or Wald tests. A multivariate logit analysis was used to further explore factors associated with using VHA care in 1996 and 1999, respectively. All analyses were adjusted for the complex survey design used in the MEPS using survey analysis techniques in STATA SE 8 [17].

## Results and Discussion

### Proportion of Veterans Using the VHA System

The percentage of veterans using the VHA system increased from 12.4% in 1996 to 14.6% in 1999 (p = 0.03). The result indicates a significant gain in VHA market share among veterans. This result is consistent with VHA data showing that the number of VHA users increased by about 17% during the same time period (2.9 million in 1996 and 3.4 million in 1999) [16].

### Characteristics of VHA Users versus Nonusers

Table 1 describes characteristics of VHA users and VHA nonusers in 1996 and 1999. In 1996, there were statistically significant differences between VHA users and nonusers in marital status, education, incomes, general and mental health status, employment status, and having usual source of care. As expected, VHA users were more likely to be not married and unemployed (12% for VHA users versus 5% for VHA nonusers) than VHA nonusers. Compared to VHA nonusers, users had lower incomes ($24,854 versus $30,222) and lower education level (26% with college or higher versus 42%). In addition, compared to VHA nonusers, more VHA users reported having fair or poor health status (29% versus 12%) as well as fair or poor mental health status (13% versus 4%). The majority of VHA users and nonusers were insured (74% versus 87%) partly because most elderly veterans (age ≥ 65) have Medicare coverage. Among veterans under age 65, VHA users were less likely to have insurance than VHA nonusers (60% versus 81%, p < 0.01). However, VHA users were more likely to have a usual source of care than non-users (87% versus 79%).

**Table 1:** Characteristics of VHA users and non-users in 1996 and 1999

	**1996**		**1999**	
**Characteristics**	**VHA Users**	**VHA Nonusers**	**VHA Users**	**VHA Nonusers**

MEPS Sample Size	534	1410	740	1234
				
Mean Age	58	57	59	57
				
%Male	94	96	93	95
				
%White	86	89	87	88
				
%Married^a^	67	76	72	72
				
%Living in the MSA Area	77	80	76	80
				
Employment Status				
%Retired	31	28	29	25
%Unemployed in the entire year ^a,b^	12	5	15	5
				
%Insured ^a,b^	74	87	81	87
%Insured for veterans < 65 years^a,b^	60	81	67	81
				
Annual Income (in 1999 dollar) ^a,b^	$24,854	$30,222	$27,593	$33,151
(standard error)	(1,429)	(875)	(1,145)	(783)
				
Education ^a^				
%Less than high school	24	16	22	20
%High school	50	51	51	50
%College degree or higher	26	33	27	30
				
Perceived Health Status^a,b,c^				
%Excellent or very good	45	65	42	61
%Good	26	23	34	28
%Fair or poor	29	12	24	11
				
Perceived Mental Health Status^a,b^				
%Excellent or very good	63	75	61	73
%Good	24	20	29	23
%Fair or poor	13	4	10	4
				
%Having Usual source of Care^a,b^	87	79	89	78

^a ^Indicates a significant difference at p < 0.05 level between users and non-users in 1996

^b ^Indicates a significant difference at p < 0.05 level between users and non-users in 1999

^c ^Indicates a significant difference at p < 0.05 level among users between 1996 and 1999

^d ^Indicates a veteran who had health insurance throughout the year

Patterns similar to those in 1996 were observed in 1999 regarding annual income, general and mental health status, employment status, insurance status, and having usual source of care. In 1999, there were no significant differences in marital status and education between VHA users and VHA nonusers.

### Characteristics of VHA Users between 1996 and 1999

In Table 1, we compare characteristics of VHA users between 1996 and 1999. There was a statistically significant difference in perceived health status among VHA users between 1996 and 1999, while the remaining characteristics were not significantly different among VHA users between the two years. Compared to 1996 VHA users, 1999 VHA users were less likely to report health status as fair or poor (29% in 1996 versus 24% in1999), and more likely to report health status as good (34% in 1999 versus 26% in 1996).

### Factors Associated with Using VHA Services

In Table 2, we summarize factors associated with using VHA services, based on logit regressions for 1996 and 1999, respectively. In 1996, age, gender, education, income, employment status, lack of insurance coverage, and perceived health status are significantly associated with the probability of using VHA care among veterans. Compared to veterans in the age group 18 – 44, the odds of using the VHA system increased with age (age 45 – 54 OR = 4.92, p < 0.01; age 55–64 OR = 4.67, p < 0.01; age ≥ 65 OR = 8.60, p < 0.01). Male veterans were more likely to use the VHA system than female veterans (OR = 20.55, p < 0.01). Veterans with high school education were more likely to use the VHA system than those with less than high school education (OR = 1.78, p < 0.01). The odds of using the VHA system significantly increased with income (OR = 1.01, p < 0.01).

**Table 2:** Significant Factors Associated with Using VHAServices by Year Based on a Logistic Analysis^1^

	**1996**		**1999**	
	
**Variable**	**Odds Ratio**	**95% CI**	**Odds Ratio**	**95% CI**
Age Group (index group = 18–44)				
45–54	4.92**	3.43–7.05	4.43**	2.81–6.98
55–64	4.67**	3.21–6.80	4.43**	2.91–6.74
≥ 65	8.60**	5.47–13.53	9.71**	6.52–14.46
				
Gender (Male = 1; Female = 0)	20.55**	12.69–33.30	15.8**	7.91–31.55
				
Race (White = 1; 0 otherwise)	1.08	0.79–1.47	1.12	0.84–1.50
				
Marital Status (Married = 1; 0 otherwise)	1.00	0.74–1.34	1.54**	1.17–2.03
				
Living in MSA (MSA = 1; 0 otherwise)	0.89	0.69–1.14	0.87	0.70–1.08
				
Education (index group = less than high school)				
High school	1.78**	1.31–2.40	1.60**	1.20–2.14
College or more	0.97	0.67–1.42	1.14	0.80–1.62
				
Income (in $1,000)	1.01**	1.00–1.02	1.00	0.99–1.01
				
Employment Status (index group = employed)				
Retired	1.70**	1.14–2.52	1.44*	1.06–1.92
Unemployed	1.68**	1.15–2.45	1.61**	1.07–2.41
				
Uninsured^2 ^(index group = insured)	2.15**	1.6–2.90	1.78**	1.27–2.50
				
Perceived health status (index group = excellent or very good)				
Good	1.42*	1.07–1.89	1.71**	1.36–2.15
Fair or poor	2.12**	1.56–2.89	2.26**	1.69–3.03
				
Perceived mental health status (index group = excellent or very good)				
Good	0.93	0.69–1.25	0.94	0.74–1.19
Fair or poor	1.30	0.87–1.96	1.04	0.70–1.55

Note: * p < 0.05; ** p < 0.01

1. The dependent variable is the VHA user status with 1 indicating VHA users and 0 indicating nonusers.

2. Indicates veterans who were uninsured throughout the year, or a portion of the year.

Compared to employed veterans, both retired and unemployed veterans were more likely to use the VHA system. The odds ratio was 1.70 for retired veterans (p < 0.01) and 1.68 for unemployed veterans (p < 0.01). Furthermore, the odds ratio of using the VHA system for uninsured veterans was 2.15 (p < 0.01) compared to insured veterans. Finally, the odds of using VHA care increased as self-reported health status worsened. Compared to veterans in excellent or very good health status, the odds ratio of using the VHA system was 1.42 (p < 0.05) for those in good health status and 2.12 (p < 0.01) for those in fair or poor health status.

Similar veteran characteristics associated with using VHA care found in 1996 were also found in 1999, with the exception of marital status and income. Marital status was not a significant factor in 1996, but married veterans were more likely to use VHA than unmarried veterans in 1999 (OR = 1.63, p < 0.01), controlling for other factors. In contrast, income was not a significant factor in 1999, while it was significantly associated with using VHA care in 1996.

To further examine the changes in characteristics between the two years, we conducted a sensitivity analysis by pooling the data from 1996 and 1999 to examine the interactions between the year indicator and individual characteristics. The analysis shows that the results are consistent with those in the separate logit regression analyses.

## Conclusion

We provided an overview of changes in health insurance status and demographic characteristics of veterans using the VHA health care system during a period when VHA implemented major organizational changes between 1996 and 1999. The VHA system provides a special safety net for a significant number of veterans that is not available to most Americans. The proportion of veterans using the VHA system increased significantly from 12.4% in 1996 to 14.6% in 1999. This pace has continued as the number of veterans treated in the VHA health care system increased by 36% between 1998 and 2002 [18]. In January 2003, VHA established a policy to stop enrolling Priority Group 7 veterans after January 16, 2003 due to the increase in enrollment and lack of resources.

The results indicate that VHA users characteristics did not change substantially following the major reorganization and policy changes. Only two variables, marital status and income, are different between the two years. Married veterans were more likely to use VHA care in 1999, but not in 1996. In addition, the odds of using VHA care increased with income in 1996, while income was not statistically significant in 1999. As a result, we reject the hypothesis that more veterans with health insurance and healthier veterans use VHA care after the major reforms of 1996. The fact that we did not observe changes in VHA user characteristics may suggest that sizeable numbers of veterans needed care but were not eligible to receive it through VHA prior to the changes in the VHA systems in 1996.

Our results suggest that being uninsured, unemployed, in poor health status remain significant factors associated with using the VHA system in the both years. Even though VHA users more likely to be uninsured than VHA nonusers, VHA users were more likely to report having a usual source of care than VHA nonusers. Therefore, VHA appears to have continued its mission as a safety net to serve vulnerable veterans who were in poor health, uninsured, or unemployed.

In addition, the findings indicate that a majority of VHA users had alternative insurance coverage, such as Medicaid, Medicare, or private health insurance. This may have important implications for quality of care and coordination of care [13]. For patients with access to VHA and other health care systems, there is an increasing need for communication between VHA and non-VHA providers to ensure continuity and quality of care. Further research on strategies of sharing information among providers in VHA and other health care systems is needed to improve care for patients using both VHA and non-VHA care.

This study has two notable limitations. The MEPS study sample, including the veteran sample, is potentially biased because the households in MEPS were recruited via mail or telephone contact from the sample of the National Health Interview Survey. Homeless veterans, a highly vulnerable group, are likely to be under-sampled because they have no permanent residence or telephone access. Second, this is a cross-sectional study rather than a cohort study. Therefore, we were unable to evaluate the discrete impact of individual policy change between 1996 and 1999. However, using the two cross-sectional MEPS samples provides a unique opportunity to examine the differences in the characteristics of VHA veteran users and non-users during the period when VHA experienced major policy and organizational changes.

In summary, the VHA health care system is the nation's largest integrated health care system, providing a special health care safety net for indigent and vulnerable veterans. We observed that characteristics of VHA users did not change following the reorganization of the VHA health care delivery system and changes in eligibility and enrollment policy. VHA appears to have continued its mission as a safety net to serve vulnerable veterans who were in poor health, uninsured, or unemployed after major organizational changes.

## Competing interests

The author(s) declare that they have no competing interests.

## Authors' contributions

CFL, MLM, and AEBS designed the conceptual framework for the study. CFL designed the method, conducted data analysis, and drafted the manuscript. CFL, MLM, and AES interpreted the results.

## References

[B1] (2002). Statistical Abstract of the United States. City: US Census Bureau.

[B2] Kizer KW, Demakis JG, Feussner JR (2000). Reinventing VA health care: systematizing quality improvement and quality innovation. Med Care.

[B3] Kizer KW, Pane GA (1997). The "new VA": delivering health care value through integrated service networks. Ann Emerg Med.

[B4] Fortney JC, Borowsky SJ, Hedeen AN, Maciejewski ML, Chapko MK (2002). VA community-based outpatient clinics: access and utilization performance measures. Med Care.

[B5] Chapko MK, Borowsky SJ, Fortney JC, Hedeen AN, Hoegle M, Maciejewski ML, VanDeusen Lukas C (2002). Evaluation of the Department of Veterans Affairs community-based outpatient clinics. Med Care.

[B6] Department of Veterans Affairs (2003). Facts about the Department of Veterans Affairs. Press Release.

[B7] Department of Veterans Affairs (1999). Resource Allocation for Veterans' Health Care. Press Release.

[B8] Department of Veterans Affairs (1997). Department of Veterans Affairs Strategic Plan, Fiscals Years 1998–2003 Office of the Assistant Secretary for Policy Planning.

[B9] Department of Veterans Affairs (2002). Medical Care Cost Recovery. Press Release.

[B10] Wilson NJ, Kizer KW (1997). The VA health care system: an unrecognized national safety net. Health Aff (Millwood).

[B11] Agha Z, Lofgren RP, VanRuiswyk JV, Layde PM (2000). Are patients at Veterans Affairs medical centers sicker? A comparative analysis of health status and medical resource use. Arch Intern Med.

[B12] Ashton CM, Souchek J, Petersen NJ, Menke TJ, Collins TC, Kizer KW, Wright SM, Wray NP (2003). Hospital use and survival among Veterans Affairs beneficiaries. N Engl J Med.

[B13] Shen Y, Hendricks A, Zhang S, Kazis LE (2003). VHA enrollees' health care coverage and use of care. Med Care Res Rev.

[B14] Cohen J (1997). Design and Methods of the Medical Expenditure Panel Survey Household Component. Agency for Health Care Policy and Research.

[B15] (1997). Statistical Abstract of United States. US Census Bureau.

[B16] (2000). Statistical Abstract of United States. US Census Bureau.

[B17] (2003). STATA Reference Manual. College Station, TX.

[B18] Department of Veterans Affairs (2003). VA Health Care, Systemwide Workload, FY1997-FY2002. http://www.va.gov//vetdata/ProgramStatics/.

